# Editorial: Xenophagy: Its role in pathogen infections

**DOI:** 10.3389/fcimb.2022.1003451

**Published:** 2022-08-26

**Authors:** Xiaona Zhao, Yongxia Liu, Hongwei Wang, Wentao Li, Jianzhu Liu

**Affiliations:** ^1^ College of Veterinary Medicine, Shandong Agricultural University, Taian, China; ^2^ College of Animal Science and Technology, Henan University of Science and Technology, Henan, China; ^3^ Department of Environmental Health Science, University of Georgia College of Public Health, Athens, GA, United States

**Keywords:** Viruses, bacteria, infection, xenophagy, pathogen

Autophagy, a lysosome-dependent catabolic pathway, exists in eukaryotic cells and involve many biological functions such as cell differentiation, starvation tolerance and immune defense. It is defined as the process of specific identification and clearance of intracellular pathogenic microorganisms by eukaryotic cells, which is an effective way for immune cells to exercise host defense (Suares et al., 2021). Autophagy can degrade relatively large substrates, such as protein aggregates, organelles and invading pathogens. Based on the different ways of autophagolysosome degradation, it can be divided into three types: molecular chaperone-mediated autophagy (CMA), microautophagy and macroautophagy. Macroautophagy is the fusion of lysosomes and vesicles containing substances to degrade a variety of intracellular components, such as peptides, organelles, intracellular protein aggregates and pathogens. The process of degrading invading pathogens through macroautophagy is also known as xenophagy (Gatica et al., 2018).

Xenophagy is a unique and selective autophagy that can resist a variety of intracellular pathogens, such as viruses, bacteria and parasites. It protects host cells from lethal damage, and plays a key role in innate immunity (Cong et al., 2020). Xenophagy, promotes macrophage clearance of cytoplasmic invaders after pathogenic DNA exposure or phagocytic membrane disruption by pathogens (Shao et al., 2022). A total of 8 papers are collected in this topic, expounding the role of xenophagy in animal viral and bacterial infections.

## Autophagy and animal virus infection

Increasing evidence suggests a link between autophagy and virus. This study summarized the interactions between autophagy and viruses in porcine, poultry, ruminants and other animals, such as Pseudorabies virus, Porcine parvovirus, Foot-and-mouth disease virus, etc (Jiang et al.). Although autophagy promotes the replication of most animal viruses, the mechanism of how virus affects autophagy is different. Liu et al. reported that DHAV-1 2B protein induces autophagy by blocking the fusion of autophagosomes with lysosomes and blocked the complete occurrence of autophagy flow (Liu et al., 2021). The virus’s inhibition of autophagic flow leads to the accumulation of autophagosomes, which may lead to the blockage of intracellular vesicle trafficking and circulation. The stacked membrane structures may provide replication sites for viruses and promote viral replication and synthesis. Studies shows that miRNAs are closely related to autophagy. The study of integrated microRNA (miRNA) and mRNA expression profiles revealed that the differential expression level of miR-222a confirmed that it could be used as an antiviral factor against DHAV-1 Infection, which may be related to the inhibition of autophagy by miRNA through regulating the activation of related pathways of downstream target genes. (Sui et al.).

In this Research Topic, another paper shows that the nonstructural protein MGF360-14L of African swine fever virus (ASFV) inhibits IFN-I production by promoting TRIM21-mediated ubiquitination of IRF3 to degrade IRF3 (Wang et al.). The ubiquitination system of host cells also plays an important role in allogeneic autophagy (Franco et al., 2017).

## Autophagy and bacteria-induced inflammation

Bacterial invasion and host cell resistance against bacterial infection by heterophagy is a process in which pathogens interact with host cells. It has been reported that Salmonella infection can cause xenophagy in host cells. As a innate immune mechanism to resist bacterial infection, xenophagy targets bacteria in the cytoplasm and damages SCV and phagosomes to limit Salmonella reproduction in host cells. Although xenophagy plays an important role in constituting the effective defense mechanism of host against invading pathogens, various intracellular bacteria have adopted different strategies to avoid the degradation of xenophagy. For example, to avoid the recognition by xenophagy, intracellular Salmonella can inhibit the xenophagy signaling pathway. Effector proteins can prevent the fusion of autophagosomes and lysosomes to avoid the degradation of Salmonella by lysosomes, and eventually cause bacterial infection (Tattoli et al., 2012). The interaction between bacterial pathogens and infected host cells determines the survival or extinction of the former. In the study on the role of LysR regulators (BSS2_II0858) in *Brucella suis* S2 infection escaping host autophagy, it was found that BSS2_II0858 gene was inactive in the process of apoptosis. Δ0858 mutant could promote the transformation of LC3-I to LC3-II, significantly inhibit the early autophagy flux, and lead to significant accumulation of autophagosomes. Therefore, BSS2_II0858 manipulates the host autophagy flux, which may be related to the survival of Brucella in macrophages (Zhang et al.).

The other study concluded that *P. gingivalis* can avoid degradation by blocking the combination of autophagosomes and lysosomes. In this case, *P. gingivalis* can colonize and proliferate in monolayer-coated vacuoles after invasion. The autophagosomes of monolayers were modified by *P. gingivalis*, and could not fuse with lysosomes to form autophagosomes(Kang et al.). *P. gingivalis* also could invade ARPE cells, escape from autophagy vesicles, enter a single membrane structure, and freely occupy the cytoplasm of ARPE cells (Arjunan et al., 2020)(Kang et al.).

To sum up, these results provide readers with updated data on the role of xenophagy in pathogens infection. While there is currently some relevant literature on this Research Topic, the papers published in this Research Topic strongly demonstrate the mechanism of Xenophagy in promoting the replication of most animal viruses and bacteria. After reading this Research Topic, readers would learn more about the role of Xenophagy in animal disease.

## Author contributions

Writing, XZ and JL; review & editing, YL, HW, WL, and JL. All authors contributed to the article and approved the submitted version.

## Funding

This projct was supported by the Natural Science Foundation of Shandong Province, China (ZR2021MC088), the National Natural Science Foundation of China (31872535 and 31802259).

## Conflict of interest

The authors declare that the research was conducted in the absence of any commercial or financial relationships that may be interpreted as a potential conflict of interest.

## Publisher’s note

All claims expressed in this article are solely those of the authors and do not necessarily represent those of their affiliated organizations, or those of the publisher, the editors and the reviewers. Any product that may be evaluated in this article, or claim that may be made by its manufacturer, is not guaranteed or endorsed by the publisher.
